# Immediate loading with fixed full-arch prostheses in the maxilla: Review of the literature

**DOI:** 10.4317/medoral.19664

**Published:** 2014-06-01

**Authors:** David Peñarrocha-Oltra, Ugo Covani, Miguel Peñarrocha-Diago, Maria Peñarrocha-Diago

**Affiliations:** 1 Master of Oral Surgery and Implantology. Department of Stomatology, Valencia University Medical and Dental School; 2 Chairman, Department of Surgery, University of Pisa, Italy; 3 Chairman of Oral Surgery, Department of Stomatology, Valencia University Medical and Dental School, Spain; 4 Full Professorof Oral Surgery, Department of Stomatology, Valencia University Medical and Dental School, Spain

## Abstract

Objectives: To critically review the evidence-based literature on immediate loading of implants with fixed full-arch prostheses in the maxilla to determine 1) currently recommended performance criteria and 2) the outcomes that can be expected with this procedure. Study Desing: Studies from 2001 to 2011 on immediate loading with fixed full-arch maxillary prostheses were reviewed. Clinical series with at least 5 patients and 12 months of follow-up were included. Case reports, studies with missing data and repeatedly published studies were excluded. In each study the following was assessed: type of study, implant type, number of patients, number of implants, number of implants per patient, use of post-extraction implants, minimum implant length and diameter, type of prosthesis, time until loading, implant survival rate, prosthesis survival rate, marginal bone loss, complications andmean follow-up time. Criteria for patient selection, implant primary stability and bone regeneration were also studied. Results: Thirteen studies were included, reporting a total of 2484 immediately loaded implants in 365 patients. Currently accepted performance criteria regarding patient and implant selection, and surgical and prosthetic procedures were deduced from the reviewed articles. Implant survival rates went from 87.5% to 100%, prosthesis survival rates from 93.8% to 100% and radiographic marginal bone loss from 0.8 mm to 1.6 mm.No intraoperative complications and only minor prosthetic complications were reported. Conclusions: The literature on immediate loading with fixed full-arch prostheses in the maxilla shows that a successful outcome can be expected if adequate criteria are used to evaluate the patient, choose the implant and perform the surgical and prosthetic treatment. Lack of homogeneity within studies limits the relevance of the conclusions that can be drawn, and more controlled randomized studies are necessary to enable comparison between the immediate and the conventional loading procedures.

** Key words:**Immediate loading, full-arch, dental implants, loading protocols.

## Introduction

Terminology regarding implant loading protocols was considered in a Consensus Meeting of the Spanish Implant Society in 2002 (1): immediate loading was considered when the prosthesis was placed on the same day of implant placement, early loading when it was placed before the conventional osseointegration period of 3 to 6 months, and delayed loading when it was placed after 3 to 6 months. Thereafter temporary boundaries between loading protocols have successively been reviewed (2,3). The last and most accepted classification was presented by Esposito *et al.* (4) in their Cochrane systematic review 2007 update: immediate loading was considered the establishment of occlusal function of implants during the first week after implant placement, early loading within one week and two months and conventional loading from two months onwards; the separate consideration of delayed loading was suppressed for being unnecessary.

Implant-supported fixed full-arch prostheses are at present the treatment alternative which best rehabilitates oral functions in edentulous patients. Classic protocols propose that implants should be unloaded during osseointegration (3 to 4 months in the mandible and 6 to 8 months in the maxilla) (5). Micromovements have been considered, since the start of implant dentistry, one of the main risk factor for osseointegration (6). Thus, edentulous patients receiving implants are classically given removable dentures during this period, which they often find uncomfortable.

Schnitman *et al.* (7) reported the first case series patients successfully rehabilitated with immediately loaded fixed prostheses. Several advantages have been related to immediate loading, including immediate function and esthetics, avoidance of temporary removable prostheses, avoidance of second surgeries and preservation of soft tissue anatomy (8). According to recent studies, implants immediately loaded with fixed full-arch prostheses achieve very high success rates after several years of follow-up, both in post-extraction and healed bone, and both in maxilla and mandible (9). However, Esposito *et al.* (10) in their last Cochrane systematic review on loading protocols concluded that in selected patients immediate loading can be successfully performed, but that tendencies indicate that immediately loaded implants fail more frequently than those loaded conventionally. Furthermore, these authors concluded that immediate loading in edentulous mandibles is well documented, but less evidence is available for the maxilla.

The aim of this report is to critically review the evidence-based literature on immediate loading of implants with fixed full-arch prostheses in the upper maxilla in order to determine 1) currently recommended performance criteria and 2) the outcomes that can be expected with this procedure.

Material and Methods

A data search was performed using PubMed’s electronic database of dental reports and reviews of clinical studies, using the following search terms in simple or multiple conjunctions: “immediate loading,” “implant loading,” “loading protocol,” “edentulous maxilla,” “full-arch.”The years searched were 2001 to 2011. Review articles and references from different studies were used to identify relevant studies.

To select the studies all obtained reports were reviewed. Titles and abstracts were screened for relevance. The full text of relevant abstracts was obtained and selected using the following inclusion and exclusion criteria.

Inclusion criteria:

• Immediate loading with fixed full-arch prostheses performed in the upper maxilla.

• Clinical series of at least 5 patients.

• Follow-up of at least 12 months after prosthetic load.

Exclusion criteria:

• Case reports.

• Studies with missing data.

• Repeatedly published studies; the last version was included.

• Studies in other languages than English or Spanish.

The initial literature search yielded 118 articles. After the first screening based on the title and abstract, 20 studies were found eligible. Full-text review of these studies determined the selection of 13 articles for analysis. One was a controlled randomized prospective study, one was a controlled non-randomized prospective study, seven were prospective single-cohort studies and five had a retrospective design.

In each study the following variables were assessed and are collected in  Table 1: type of study, implant type, number of patients, number of implants, number of implants per patient,use or not of immediate post-extraction implants, minimum implant length and diameter, type of fixed immediately loaded prosthesis, time until loading, implant survival rate, prosthesis survival rate, peri-implant marginal bone loss and mean follow-up time. Furthermore, criteria for patient selection, adequate implant primary stability and bone regeneration procedures were also studied.

**Table 1:**
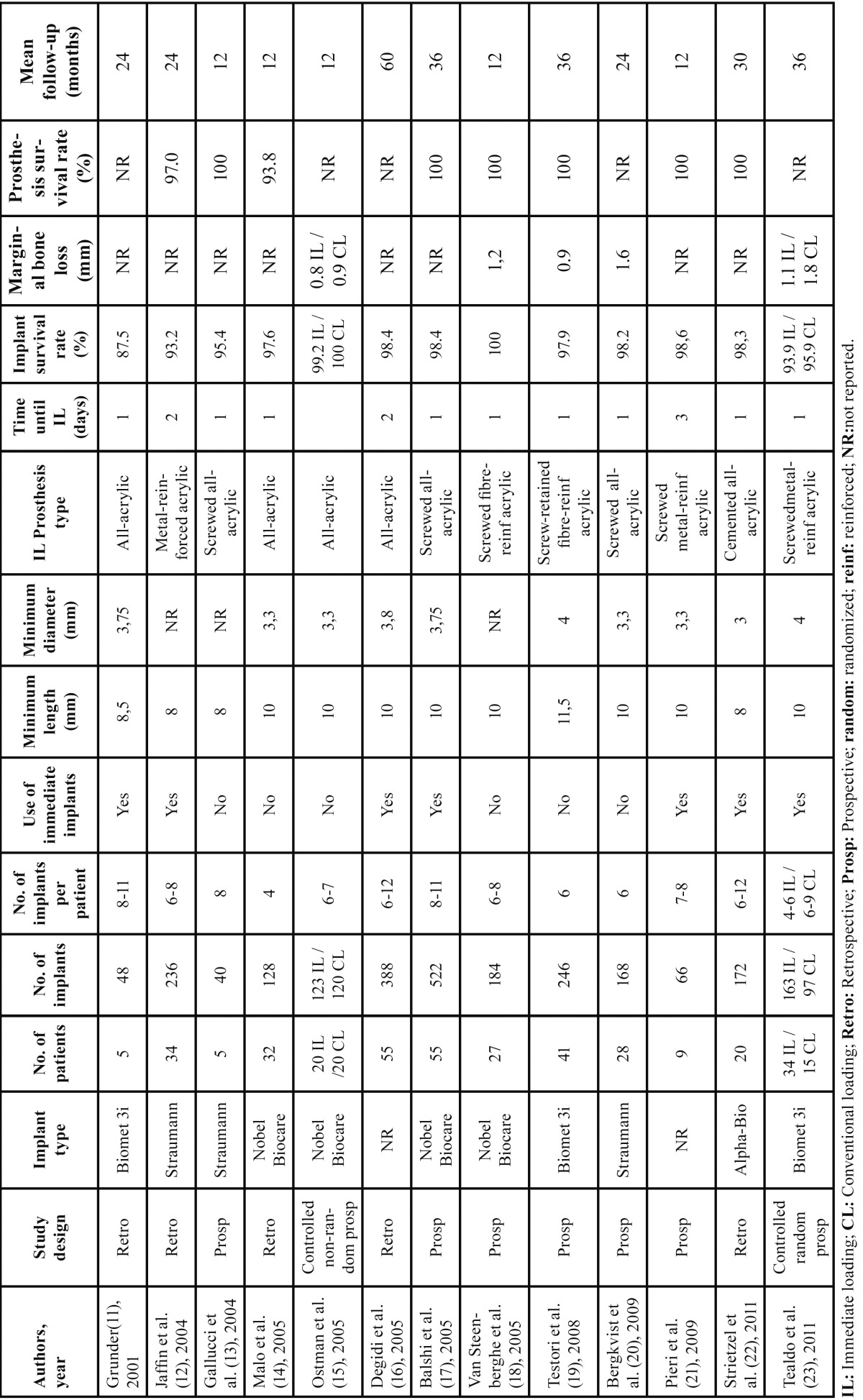
Studies on immediate loading with fixed full-arch prostheses in the upper maxilla.

## Results

Thirteen studies fulfilled the selection criteria,which reported a total of 2484 immediately loaded implants using fixed full-arch maxillary prostheses in 365 patients ( Table 1 (11-23)).

▪Performance criteria

-Patient selection

Criteria to select patients for immediate loading may influence the achievement of predictable results. Most studies propose the following criteria to perform full-arch immediate loading: good health, edentulous ma-xilla or teeth with impossible prognosis, enough bone quantity and quality, absence of acute infection, and primary stability of implants. Many criteria are also used to exclude patients: systemic disease, inmunodeficiencies, head and neck radiotherapy, alcohol or drug abuse, pregnancy, pathologies of the oral mucosa, or lack of cooperation of the patient (11-23). Diabetes under control is considered not to be an exclusion factor for immediate loading (11-13,16).

There is no consensus on bruxism or smoking habits. Some studies do not include bruxists (16,19,21) or smokers of over 10 cigarettes per day (13,21). Grunder *et al.* (11) treated 8 patients of which 4 showed clear signs of bruxism; 5 of the 7 failed implants happened inbruxist patients. Jaffin *et al.* (12), on the contrary, included bruxists and obtained a high implant survival rate (12). Degidi *et al.* (16) treated 43 patients, of which 15 were smokers, and found no relationship between implant survival (98% after 5 years) and smoking. In the study of Grunder *et al.* (11) 6 out of 8 patients smoked up to 50 cigarettes per day and no influence on implant survival was found.

-Primary stability

All reviewed studies agreed in regarding initial implant stability as the key requirement for the success of immediate loading (11-23).Most studies establish a minimum implant insertion torqueto perform immediate loading, which variesfrom 30 to 45 Ncm (8,21,23,24); some studies evaluate primary stability using an Ostell ISQ® device and require an ISQ of at least 60 (8). If enough implants are placed, immediate loading can be performed even if not all the implants achieve an adequate stability; unstable implants should be left unloaded (21).

Some studies suggest procedures to enhance primary stability, such as 1-2 mm subcrestal implant placement (8,20,24), bicorticalization into the nasal or sinus floor whenever possible (14) and implant site underpreparation (21,23).

-Implant number and position

Most studies consider 6 implants to be the lowest adequate number to achieve a predictable outcome (12,16,18-20,22). Other authors use a minimum of 8 (11,17), and some use up to 12 implants (16,22). Degidi *et al.* (16) used 6 to 12 implants (average 9), and after 5 years found a higher survival rate when ≤ 10 implants were placed (99.2%) than with > 10 (96.3%; *p*=0.033). On the contrary, Balshi *et al.* (17) found no relationship between number of implants and implant success. Malo et al. (14) described a technique, subsequently supported by other authors (23), which achieves successful results with fixed prostheses over 4 implants. However, according to the IV ITI Consensus Conference, this procedure is only clinically well documented with 6 or more implants and scientific evidence using 4 implants is scarce (9).

Regarding implant position, authors give importance to a uniform distribution along the alveolar arch; depending on the number of implants used, distal implants emerge in 2nd premolar or 1st molar position (12,20). In some studies distal implants are placed with tilted orientation, enabling the placement of longer implants and minimizing the need of cantilevers (19,23).

-Implant length and diameter

Kinsel *et al.* (25) studied factors generally considered of risk for immediately loaded implants, and a reduced length was the only statistically significant factor. Most authors consider 10 mm (17,23) to be the minimum adequate implant length to predictably perform immediate loading with full-arch prosthesis in the maxilla. Other studies use some 8 mm implants combined with other longer implants (11,12). However, all authors prefer using longer implants whenever possible, being 13 and 15 mm implants the most frequently used (17,23). Tilting may enable placement of longer implants in posterior regions (19,23).

Regarding implant diameter, 4 mm implants are usually the first choice, while wider implants are used in posterior regions with poor quality bone (17,20,23). Some studies (11,16) have reported higher failure rates with wide diameter implants; however, this is associated with placement in class III-IV bone. The use of narrow implants (up to 3.0 mm) has also been reported; however, only whenever strictly necessary and long implants (13-15 mm), splinted to other wider implants, are used (14,15,20-22).

-Bone regeneration procedures

According to the ITI Consensus Conference in 2008, conventional loading should be the first choice whenever sinus lift and/or reconstruction of the alveolar ridge are performed (26). Many studies on immediate loading do not use bone grafts, not even to cover small peri-implant defects or fill in the jumping distance gap in post-extraction implants (8,11,14,15,18,19). However, some of the most recent studies reported the successful use of autologous or bovine bone to cover dehiscences and fenestrations or fill in the horizontal gap between implant and socket walls (21,25).

-Prosthetic procedures

Several techniques have been reported to produce temporary immediate fixed full-arch prostheses, but it can be summarized into: 1) clinical adaptation of a previously fabricated denture/prosthesis which is delivered immediately after implant placement (17,22), or 2) intraoperative register of implant positions and production of the temporary fixed prosthesis by the dental technician that will be delivered within the first week (11,12,21). With independence of the fabrication technique, immediate loading prostheses may be cemented or screw-retained ( Table 1 (11-23)). Clinically-adapted prostheses are often all-acrylic non-reinforced. Grunder et al. (11) reported negative results with these prostheses, 3 of which fractured and had to be repaired; 5 of the 7 failed implants occurred in those 3 cases. Other authors have favorably used all-acrylic prostheses (14,17,20). When the prosthesis is produced by the dental technician the acrylic can be more easily reinforced. According to Tealdo *et al.* (23), reinforcement can be a key factor for the success of immediately loaded implants because it minimizes micromovements during osseointegration. Many studies use metal-reinforcement, which can be cast (12,23) or CAD/CAM-produced (21); other studies have used fiberglass-reinforced prostheses with good results (18,19).

With respect to prosthetic design, the avoidance of distal cantilevers is considered key by many authors (8,17,21,25). A thorough occlusal adjustment that ensures a uniform load distribution is also important (11,21,23). Moreover, most studies recommend a soft diet during osseointegration (8,16,17,21).

▪Outcomes

-Implant survival

Only 2 controlled studies have compared the outcome of immediate and conventional loading with fixed full-arch prostheses in the maxilla (15,23). Ostman *et al.* (15) performed a prospective non-randomized study including 20 patients per group, and 123 immediately loaded and 120 conventionally loaded implants; survival rates were 99.2% and 100% respectively. Tealdo *et al.* (23) randomly divided 49 patients and treated 34 with immediate loading and 15 with conventional loading; 163 implants were immediately loaded and 97 conventionally loaded; after 36 months, 10 and 4 implants had failed respectively, yielding 93.9% and 95.9% survival rates, which were not significantly different. Several other studies have reported high implant survival and success rates with immediately loaded fixed full-arch prostheses ( Table 1 (11-14,16-22)). Degidi *et al.* (16) and Balshi *et al.* (17) presented the largest samples and the longest follow-up times. Degidi *et al.* (16) studied 388 implants and after 5 years 6 had been lost (98.4% survival rate), all of them during the first 6 months. Balshi *et al.* (17) treated 55 patients with 522 implants and achieved a 98.4% success rate after an average follow-up of 3 years. Only one study (11) presented a survival rate of less than 90% (87.5%); however, this was a short series of only 5 patients.

Seven of the included studies performed immediate loading on immediate post-extraction implants, and reported similarly high survival rates as those using only implants placed into healed sites ( Table 1 (11,12,16,17,21-23)). In the study by Degidi *et al.* (16), 213 of the 388 immediately loaded implants were immediate post-extraction; after 5 years survival rates were 98.1% and 98.9% for immediate and non-immediate implants respectively, being differences non statistically significant. Pieri *et al.* (21) detailed that 9 patients were treated with both post-extraction implants (n=24) and implants placed in healed sites (n=42); only 1 implant failed in each group, yielding success rates of 95.8% and 97.6%.

-Prosthesis survival

Several publications have studied the outcome of temporary prostheses used for immediate loading. Most report 100% survival rates, despite the loss of one or more implants in some patients (11,13,15-22). Only Jaffin *et al.* (12) substituted one fixed prosthesis for a removable denture during osseointegration due to the loss of several implants. Malo *et al.* (14) and Tealdo *et al.* (23), on the contrary, used rescue implants to avoid having to substitute fixed prostheses for dentures.

-Marginal bone loss

Only 2 of the reviewed studies analyzed differences in peri-implant bone loss between immediately and conventionally loaded implants (15,23). Tealdo *et al.* (23) used intraoral radiographs obtained with a long-cone paralleling technique, individual film holder and a customized centric occlusion registrations to assess bone loss after 12, 24 and 36 months. Bone loss was significantly lower for the immediate loading group at the 3 time points: after 12 months it was 0.8± 0.8 for the immediate loading and 1.4 ± 0.8 for the conventional loading group; after 24 months, 1.0 ± 0.9 and 1.7 ± 0.9; and after 36 months, 1.1 ± 0.9 and 1.8 ± 1.1 respectively. Ostman *et al.* (15) obtained an average 0.8 ± 0.9 bone loss for immediately loaded and 0.9 ± 1.0 for conventionally loaded implants; differences were not significant.

Other authors have evaluated bone loss around immediately loaded implants with fixed full-arch prostheses and values reported vary between 0.8 and 1.6 mm. Only one study, by Pieri *et al.* (21), analyzed differences between immediately loaded immediate post-extraction (0.6 ± 0.3 mm) and non-immediate implants (0.5 ± 0.2); however, data in this study was not detailed between maxilla and mandible.

-Complications

Both biological and prosthetic complications are rare in studies on immediate loading with full-arch prostheses. None of the stud-ies reported intraoperative complications, and the only postoperative complications were swelling and pain, generally light and only in some case severe (16,25,26). Other reported biological complications, although not directly related to immediate loading, were mucositis and periimplantitis; Van Steenberghe *et al.* (18) found mucositis in 4 patients and periimplantitis in 1 at the 12-month follow-up visit, which were treated and solved.

The most frequently reported complications were those related with the temporary immediately loaded prostheses; most were minor and could be easily solved. Van Steenberghe *et al.* (18) found a broken screw and occlusal fractures in 2 patients. The only complication in the study by Pieri *et al.* (21) was the fracture of an acrylic tooth which happened in 3 cases. The most severe complications were those that lead to implant failures: Grunder (11) and Malo *et al.* (14) described fractures in all-acrylic prostheses that caused the loss of implants due to harmful forces.

## Conclusions

The literature on immediate loading with fixed full-arch prostheses in the maxilla shows that a successful outcome can be expected if adequate criteria are used to evaluate the patient, choose the implant and perform the surgical and prosthetic treatment. High implant and prosthetic survival rates, low marginal bone loss and few complications are reported by the studies on the topic. However, studies available use different surgical procedures, types of prostheses, loading times and have very different study designs. This lack of homogeneity limits the relevance of the conclusions that can be drawn. Furthermore, more controlled randomized studies are necessary to enable scientific comparison of the immediate loading and the conventional loading procedures.
